# An Asp49 Phospholipase A_2_ from Snake Venom Induces Cyclooxygenase-2 Expression and Prostaglandin E_2_ Production via Activation of NF-**κ**B, p38MAPK, and PKC in Macrophages

**DOI:** 10.1155/2014/105879

**Published:** 2014-04-06

**Authors:** Vanessa Moreira, Bruno Lomonte, Marco Aurélio Ramirez Vinolo, Rui Curi, José María Gutiérrez, Catarina Teixeira

**Affiliations:** ^1^Laboratório de Farmacologia, Instituto Butantan, Avenida Vital Brazil 1500, 05503-900 São Paulo, SP, Brazil; ^2^Instituto Clodomiro Picado, Facultad de Microbiología, Universidad de Costa Rica, San José, Costa Rica; ^3^Departamento de Genética, Evolução e Bioagentes, Instituto de Biologia, Universidade Estadual de Campinas, Campinas, SP, Brazil; ^4^Departamento de Fisiologia, Instituto de Ciências Biomédicas, Universidade de São Paulo, São Paulo, SP, Brazil

## Abstract

Phospholipases A_2_ (PLA_2_) are key enzymes for production of lipid mediators. We previously demonstrated that a snake venom sPLA_2_ named MT-III leads to prostaglandin (PG)E_2_ biosynthesis in macrophages by inducing the expression of cyclooxygenase-2 (COX-2). Herein, we explored the molecular mechanisms and signaling pathways leading to these MT-III-induced effects. Results demonstrated that MT-III induced activation of the transcription factor NF-**κ**B in isolated macrophages. By using NF-**κ**B selective inhibitors, the involvement of this factor in MT-III-induced COX-2 expression and PGE_2_ production was demonstrated. Moreover, MT-III-induced COX-2 protein expression and PGE_2_ release were attenuated by pretreatment of macrophages with SB202190, and Ly294002, and H-7-dihydro compounds, indicating the involvement of p38MAPK, PI3K, and PKC pathways, respectively. Consistent with this, MT-III triggered early phosphorylation of p38MAPK, PI3K, and PKC. Furthermore, SB202190, H-7-dihydro, but not Ly294002 treatment, abrogated activation of NF-**κ**B induced by MT-III. Altogether, these results show for the first time that the induction of COX-2 protein expression and PGE_2_ release, which occur via NF-**κ**B activation induced by the sPLA_2_-MT-III in macrophages, are modulated by p38MAPK and PKC, but not by PI3K signaling proteins.

## 1. Introduction

PLA_2_s play key roles in numerous cellular processes in physiological and pathological conditions by regulating the release of arachidonic acid (AA), a precursor of important lipid mediators [1]. Secretory phospholipases A_2_ (sPLA_2_s) constitute a superfamily of enzymes classified into various groups (IB, IIA, IIC, IID, IIE, IIF, III, V, and X) on the basis of their source, amino acid sequence, and biochemical characteristics. Among them, group IIA sPLA_2_ includes mammalian inflammatory-type and viperid snake venom sPLA_2_ [2]. A group IIA Asp49 sPLA_2_, named myotoxin-III (MT-III), isolated from* Bothrops asper* snake venom [3], has been shown to promote marked local inflammatory events in several experimental models [4–7]. Some of these events are induced by inflammatory mediators, such as eicosanoids, produced by inflammatory cells [4]. In addition, we previously showed that this sPLA_2_ is capable of inducing cyclooxygenase-2 (COX-2) protein expression and stimulating AA and prostaglandin (PG)D_2_, PGE_2_ production, when incubated with macrophages in culture [8]. Despite the importance of prostanoids in the regulation of inflammatory events induced by sPLA_2_s, and the relevance of macrophages in this response, the signal transduction pathways that lead to MT-III-promoted biosynthesis of PGs and COX-2 expression in macrophages are unknown.

PGE_2_ is synthesized by both the constitutively expressed COX-1 and the inducible COX-2 enzymes. COX-1 is present in most tissues [9] and is responsible for generating PGs for diverse physiological and pathological functions [10]. COX-2, in turn, can be constitutively expressed in some tissues but, normally, is inducible under inflammatory conditions in several types of cells [11–14]. This expression is regulated at both the transcriptional and posttranscriptional levels. The promoter region of the COX-2 gene contains several binding sites for transcription factors including NF-*κ*B, CREB, C/EBP, and Ap-1 [13, 15, 16]. Of these, NF-*κ*B is the main transcription factor involved in COX-2 gene expression in macrophages during inflammatory processes [17, 18]. The involvement of NF-*κ*B in COX-2 expression and PGE_2_ production induced by group IIA Asp49sPLA_2_s is unknown, stressing the need to perform studies on this matter. Furthermore, it has been demonstrated that COX-2 expression correlates with the activities of intracellular signaling proteins such as p38 mitogen-activated protein kinase (p38MAPK) [19, 20], phosphoinositide 3-kinase (PI3K) [21, 22], and protein kinase C (PKC) [21, 23] in macrophages activated by several stimuli. However, the roles of these kinases in group IIA sPLA_2_-induced COX-2 expression have not been yet investigated in macrophages.

Since the production of lipid mediators is highly regulated by a variety of extracellular stimuli, it is relevant to study how the sPLA_2_s target their action to generate PGs, especially with regard to the expression of COX-2, a major isoform responsible for the production of PGE_2_ in inflammatory conditions. In this study the mechanisms by which the sPLA_2_MT-III activates macrophages leading to expression of COX-2 and release of PGE_2_ were investigated, with focus on the involvement of NF-*κ*B and the signaling pathways proteins p38MAPK, PI3K, and PKC.

## 2. Materials and Methods

### 2.1. Reagents

PGE_2_ enzyme immunoassay kits and rabbit polyclonal anti-murine COX-2 antibodies were purchased from Cayman Chemical (Ann Arbor, MI, USA); mouse monoclonal anti-rat *β*-actin antibody was from Sigma Aldrich Co. (St. Louis, MO, USA); peroxidase-conjugated secondary sheep anti-mouse or donkey anti-rabbit antibodies were from GE Healthcare (Buckinghamshire, UK). SN50, SB202190, Ly294002, H7-dihydro were purchased from Calbiochem-Novabiochem (La Jolla, CA, USA). Antibodies against phospho-p38MAPK, p38MAPK, phospho-PI3K, PI3K, and phospho-PKC were from Cell Signaling Technology (Danvers, MA). Antibody against PKC was from Santa Cruz Biotechnology (Santa Cruz, CA). RPMI 1640 and TPCK were purchased from Sigma Aldrich. Ethanol grade p.a. was obtained from Merck (Darmstadt, Germany). The salts used were purchased from Merck, GE Healthcare and Bio-Rad (Hercules, CA).

### 2.2. Animals

Male Swiss mice (18–20 g) were used. Animals were housed in temperature-controlled rooms, with a relative humidity of 65.3 ± 0.9% and 12 h dark-light period, and received water and food* ad libitum*. The animals and research protocols used in this study followed the guidelines of the Ethical Committee for Use of Animals of Instituto Butantan, SP, Brazil (CEUAIB, Protocol number 592/09), and international policies of experimental animal care. All efforts were made to minimize the number of animals used and their suffering.

### 2.3. Phospholipase A_2_ (MT-III)

MT-III was isolated from* Bothrops asper* venom by ion-exchange chromatography on CM-Sephadex C-25 using the conditions described by Lomonte and Gutiérrez [24], followed by RP-HPLC on a C8 semipreparative column (10 × 250 mm; Vydac) eluted at 2.0 mL/min with a 0–70% acetonitrile gradient containing 0.1% (v/v) trifluoroacetic acid, during 30 min, on an Agilent 1200 instrument monitored at 215 nm. Homogeneity of the final preparation was assessed by analytical RP-HPLC on a C4 column (4.6 × 150 mm) using a 0–60% acetonitrile gradient. The absence of endotoxin contamination in the MT-III preparation was demonstrated by the quantitative* Limulus* amebocyte lysate (LAL) test [25], which revealed undetectable levels of endotoxin (<0.125 EU/mL).

### 2.4. Resident Peritoneal Macrophages Collection and Culture

Resident peritoneal macrophages were harvested by washing the peritoneal cavity with 2 mL of apyrogenic saline solution. Aliquots of the washes were used to count total cell numbers in a Neubauer chamber after dilution (1 : 20, v/v) in Turk's solution. For adhesion, aliquots of either 1 × 10^6^ or 3 × 10^6^ cells/mL were added to 24- and 6-well polystyrene culture plates, respectively, and incubated for 3 h, in RPMI 1640 medium supplemented with 1% of L-glutamine and 100 *μ*g/mL of garamicine, at 37°C and 5% CO_2_ atmosphere. Nonadherent cells were removed by vigorous washing three times with glutamine-free RPMI 1640. By this procedure, peritoneal cells, which were initially composed of 40–50% of F4/80 positive cells and more than 30% of CD19 positive cells, became enriched in F4/80 positive cells (more than 90% of the adhered cells). MT-III (0.4 *μ*M) was added to macrophages in culture. This concentration was previously shown as noncytototoxic but stimulatory of macrophages functions [5, 8, 26]. At selected time intervals (0.5, 1, and 4.5 h), the plates were centrifuged at 500 g for 6 min at 22°C. The predominance of macrophages, constituting more than 95% of cells in the washes, was confirmed by light microscopic analysis of smears stained with Hema^3^ (Fisher Scientific Company, Middletown, VA). Where appropriate, the following inhibitors were used: 2.5 *μ*M TPCK (N-*α*-tosyl-L-phenylalanine chloromethyl ketone arachidonyl trifluoromethyl ketone) and 50 *μ*g/mL of SN50, selective inhibitors of NF-*κ*B activation; 1 *μ*M SB202190, an inhibitor of p38MAPK; 25 *μ*M Ly294002, an inhibitor of PI3K; 20 *μ*M H7-Dihydro, an inhibitor of PKC. All the above inhibitors were added 60 min before stimulation of macrophages with MT-III or RPMI (control). Cells treated with either inhibitors or MT-III or both were analyzed for viability by the tetrazolium-based (MTT) colorimetric assay. No significant changes in cell viability were registered with any of the above agents or the vehicle at the concentrations used (data not shown).

### 2.5. Quantification of PGE_2_ Concentration

Concentration of PGE_2_ was determined by enzyme immunoassay using commercial kits. The extraction of the prostaglandin was performed on Sep-Pak C18 columns (Waters Corporation, Milford, MA) and eluted with ethanol. In brief, 50 *μ*L aliquots of each extracted sample were incubated with the eicosanoids conjugated with acetylcholinesterase and the specific rabbit antiserum in 96-well plates were coated with anti-rabbit IgG mouse monoclonal antibody. After addition of the substrate, the absorbance of the samples was recorded at 405 nm in a microplate reader (Labsystems Multiskan), and concentrations of PGE_2_ were estimated from standard curves.

### 2.6. Western Blotting

COX-2 proteins were detected in peritoneal leukocytes or in cultured macrophages by Western blotting. Aliquots of 1 × 10^6^ cells were lysed with 100 *μ*L of sample buffer (0.5 M Tris-HCl, pH 6.8, 20% SDS, 1% glycerol, 1 M *β*-mercaptoethanol, and 0.1% bromophenol blue) and boiled for 10 min. The samples were subjected to SDS-polyacrylamide gel electrophoresis (SDS-PAGE) on 10% bisacrylamide gels overlaid with a 5% stacking gel. Proteins were then transferred to nitrocellulose membrane (GE Healthcare, Buckinghamshire, UK) using a Mini Trans-Blot (Bio-Rad Laboratories, Richmond, CA, USA). The membrane was blocked for 1 h with 5% w/v nonfat dry milk in Tris-Buffered Saline-Tween 20 (TTBS) (20 mM Tris, 100 mM NaCl, and 0.5% Tween 20) and incubated with primary antibodies against COX-2, COX-1 (1 : 1500 and 1 : 500, resp.), and *β*-actin (1 : 2000). For the study of expression and activation of protein kinases by MT-III, the membrane was blocked for 1 h in 5% w/v BSA in TTBS and incubated with antibodies against either phospho-p38MAPK, p38MAPK, phospho-PI3K, PI3K, or phospho-PKC, and PKC at 4°C with gentle shaking, overnight. The membrane was then washed and incubated with appropriate secondary antibody conjugated to horseradish peroxidase. Detection was accomplished using the enhanced chemiluminescence method according to instructions of the manufacturer (GE Healthcare, Buckinghamshire, UK). Densities of the bands were determined by a GS 800 Densitometer (Bio-Rad Laboratories, Richmond, CA) using the image analysis software Quantity One (Bio-Rad Laboratories, Richmond, CA).

### 2.7. Electrophoretic Mobility Shift Assay (EMSA)

NF-*κ*B binding capacity was assessed by EMSA. Nuclear extracts from peritoneal adherent cells (3 × 10^6^ cells/well) were obtained as previously described [27], and protein concentration was determined according to the Bradford method [28]. NF-*κ*B binding capacity was evaluated as previously described [29]. Briefly, end-labeled [*γ*-32P] ATP oligonucleotides containing an NF-*κ*B consensus-binding site (5′-AGTTGAGGGGACTTTCCCAGGC-3′) were incubated for 20 min at room temperature with 5 *μ*g of nuclear extract protein. DNA-protein complexes were then separated on a 5.5% nondenaturing polyacrylamide gel using a running buffer of 45 mM Tris, 45 mM borate, and 1 mM EDTA buffer. The gels were vacuum-dried (80°C) and subjected to autoradiography. The blots were analyzed by scanner densitometry (STORM 840, Dynamic Molecular, Sunnyvale, CA, USA). Results are expressed relative to the control condition (unstimulated control).

### 2.8. Statistical Analysis

Results are expressed as mean ± SEM. Differences among groups were analyzed by one-way analysis of variance (ANOVA) followed by Tukey's test or by the Student's *t*-test. Values of probability lower than 5% (*P* < 0.05) were considered significant.

## 3. Results

### 3.1. MT-III Activates NF-*κ*B in Isolated Macrophages

**Figure 1:**
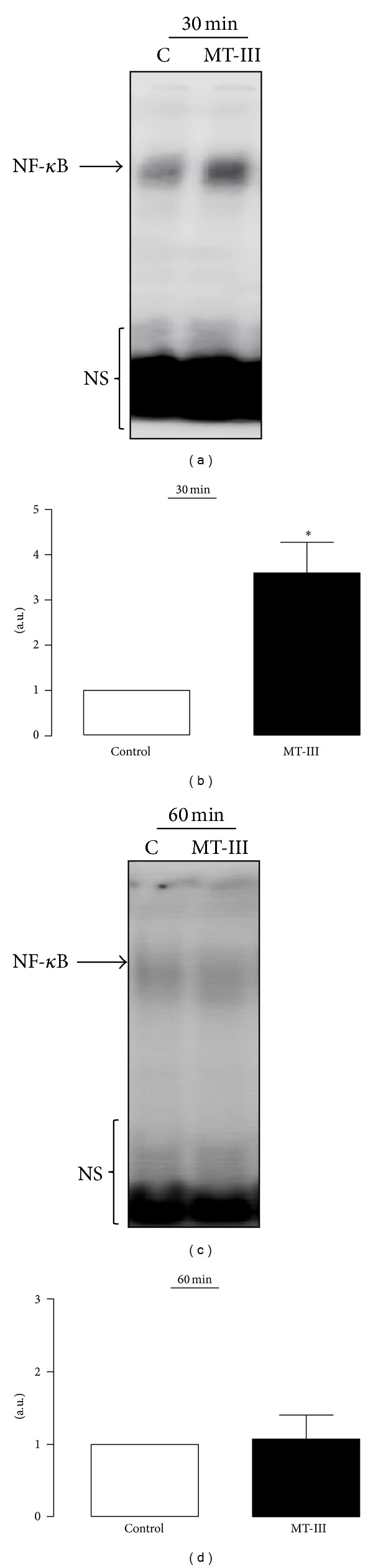
MT-III activates NF-*κ*B in macrophages in culture. (a, c) Macrophages nuclear extracts were prepared and assayed for *κ*B probe activity with ^32^P-labeled double-stranded oligonucleotide *κ*B by EMSA. (b, d) Densitometric analysis of NF-*κ*B band intensities. Results are expressed as mean ± SEM from three experiments. *P < 0.05 as compared with control value. NS: nonspecific band; C: control; NC: negative control.

Initially, it was verified whether MT-III induces activation of NF-*κ*B in peritoneal isolated macrophages. As demonstrated in Figures 1(a) and 1(b), a rapid activation of NF-*κ*B was induced by MT-III since a marked nuclear activation was detected at 30 min of incubation as compared with control cells. This is a rapid event since after one hour of incubation with MT-III, neither NF-*κ*B activation nor DNA binding was observed (Figures 1(c) and 1(d)).

### 3.2. NF-*κ*B Is Involved in COX-2 Expression and PGE_2_ Production Induced by MT-III in Isolated Macrophages

**Figure 2:**
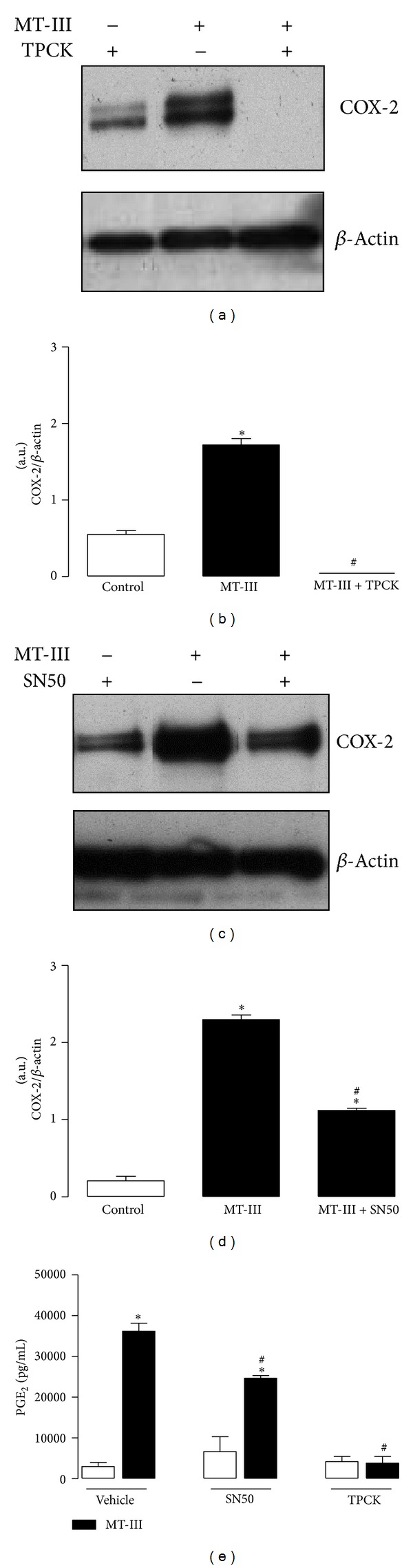
NF-*κ*B is involved in COX-2 expression and PGE_2_ release induced by MT-III in macrophages. Resident peritoneal macrophages (1 × 10^6^ cells) were pretreated with TPCK (2.5 *μ*M) or SN50 (50 *μ*g/mL) and incubated during 4.5 h with MT-III (0.4 *μ*M). (a, c) Western blotting of COX-2 and *β*-actin (loading control) of cells pretreated with SN50 or TPCK. (b, d) Densitometric analysis of immunoreactive COX-2 band intensities. (e) PGE_2_ was quantified in culture supernatants by enzyme immunoassay (see Section 2). Results are expressed as mean ± SEM from 3 experiments. *P < 0.05 as compared with control value.

The participation of the transcription factor NF-*κ*B on COX-2 protein expression and PGE_2_ production induced by MT-III was investigated using specific inhibitors of this pathway. The compound TPCK, which prevents the activation of NF-*κ*B, abrogated both COX-2 expression (Figures 2(a) and 2(b)) and PGE_2_ production (Figure 2(e)) in MT-III-stimulated macrophages. Pretreatment of cells with SN50, a cell permeable peptide that competes specifically with NF-*κ*B subunit p50 for the translocation from the cytosol into the nucleus [30], significantly reduced MT-III-induced COX-2 protein expression (Figures 2(c) and 2(d)) and PGE_2_ production (Figure 2(e)) in resident macrophages by 50% and 30%, respectively. Taken together, our data demonstrate that NF-*κ*B is involved in both COX-2 protein expression and PGE_2_ production induced by MT-III in isolated macrophages.

### 3.3. MT-III Promotes p38MAPK, PI3K, and PKC Phosphorylation in Isolated Peritoneal Macrophages

**Figure 3:**
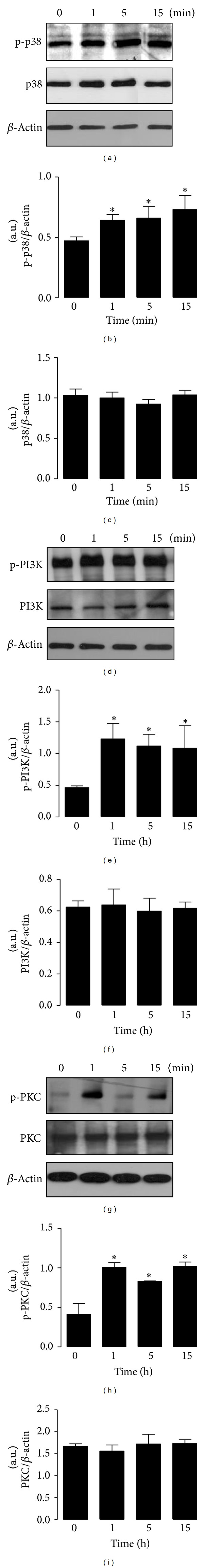
p38MAPK, PI3K, and PKC phosphorylation induced by MT-III in isolated peritoneal macrophages. Resident peritoneal macrophages (1 × 10^6^ cells) were incubated during 1, 5, and 15 min with MT-III (0.4 *μ*M). (a, d, and g) Western blotting of p-p38MAPK, p38MAPK, p-PI3K, PI3K, p-PKC, PKC, and *β*-actin (loading control). (b, c, e, f, h, and i) Densitometric analysis of immunoreactive band intensities. Results are expressed as mean ± SEM from 3 experiments. *P < 0.05 as compared to time 0.

We next verified whether MT-III causes phosphorylation in kinases that activate important signaling pathways for macrophages function. As shown in Figures 3(a), 3(d), and 3(g), unstimulated macrophages showed a basal phosphorylation on all kinases investigated. Treatment of isolated macrophages with 0.4 *μ*M MT-III resulted in a 3- to 5-fold time-dependent increase in p38MAPK, PI3K, and PKC phosphorylation over the corresponding control cells p38 (Figures 3(a) and 3(b)) and PI3K (Figures 3(d) and 3(e)) phosphorylation was detectable as early as one min and was sustained at least for 15 min. PKC phosphorylation was also detectable and peaked at one min and remained detectable at 15 min after MT-III addition (Figures 3(g) and 3(h)). Altogether, the above data demonstrate that MT-III rapidly activates phosphorylation of protein kinases in macrophages, without altering total p38, PI3K, and PKC (Figures 3(c), 3(f), and 3(i)).

### 3.4. Effect of Inhibition of Protein Kinases on PGE_2_ Production, COX-2 Expression, and NF-*κ*B Activation Induced by MT-III

**Figure 4:**
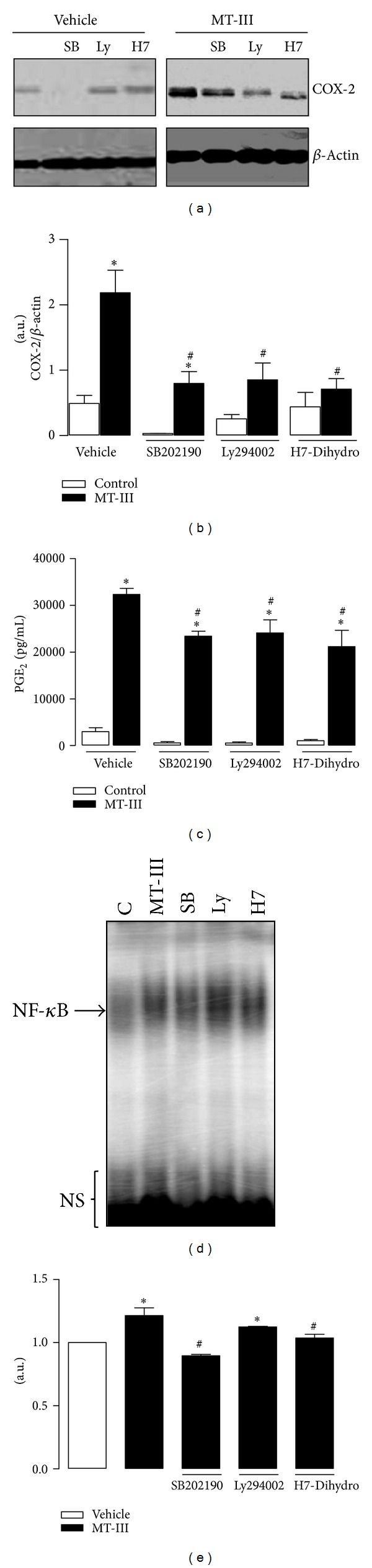
Effect of inhibition of p38MAPK, PI3K, and PKC on COX-2 expression, PGE_2_ production, and NF-*κ*B activation induced by MT-III. Resident peritoneal macrophages (1 × 10^6^ cells) were pretreated with either SB202190 (1 *μ*M), Ly294002 (25 *μ*M), or H7-dihydro (20 *μ*M) and incubated during 4.5 h with MT-III (0.4 *μ*M). In electrophoretic mobility shift assay (EMSA), resident macrophages (3 × 10^6^ cells) were pretreated with kinases inhibitors and incubated during 30 min with MT-III (a) Western blotting of COX-2 and *β*-actin (loading control). (b) Densitometric analysis of immunoreactive COX-2 band intensities. (c) PGE_2_ was quantified in culture supernatants by enzyme immunoassays (see Section 2). (d) Nuclear extracts were prepared and assayed for *κ*B probe activity with ^32^P-labeled double-stranded oligonucleotide *κ*B using EMSA. (f) Densitometric analysis of NF-*κ*B band intensities. Results are expressed as mean ± SEM from 3 experiments. *P < 0.05 as compared with control values. NS: nonspecific band; C: control.

It has been previously reported that protein kinases participate in the signaling under group IIA sPLA_2_s stimuli [31, 32]. To assess the role of kinases in the described actions of MT-III, we determined the effects of the specific inhibitors of p38MAPK, PI3K, and PKC (SB202190, Ly294002, and H7-Dihydro, resp.) on MT-III-stimulated PGE_2_ release in macrophages. MT-III-induced COX-2 protein expression (Figures 4(a) and 4(b)) and increments in PGE_2_ (Figure 4(c)) by macrophages were inhibited by SB202190, Ly294002, and H7-dihydro when compared to macrophages after 4.5 h of treatment with MT-III and pretreated with vehicle. Unstimulated macrophages showed a weak basal COX-2 protein expression when pretreated with or without inhibitors of kinases (Figures 4(a) and 4(b)). We have previously shown that resident macrophages have enhanced MT-III-induced COX-2 and PGE_2_ production dependent on NF-*κ*B activation [33]. Next, to determine whether the activation of this transcription factor is mediated by kinase signaling pathways, we tested the effect of specific inhibitors of kinases on NF-*κ*B activation by MT-III. Pretreatment of resident macrophages with SB202190 and H7-dihydro, but not Ly294002, completely inhibited MT-III-induced activation of NF-*κ*B. Taken together, these results suggest that MT-III-stimulated COX-2 expression, PGE_2_ synthesi,s and NF-*κ*B activation are mediated through the activation of distinct protein kinases, such as p38MAPK and PKC pathways.

## 4. Discussion

In this study we examined the effect of the Asp49 sPLA_2_ MT-III, isolated from* Bothrops asper* snake venom, on macrophage activation and the mechanisms through which it stimulates COX-2 expression and PGE_2_ production. Several lines of evidence clearly established that NF-*κ*B regulates the expression of several inflammatory mediators and enzymes [34]. The data shown herein demonstrate that MT-III activates NF-*κ*B. We also show that this pathway is important for COX-2 expression and PGE_2_ release in response to this toxin since incubation of macrophages with the inhibitor of I*κ*B phosphorylation (TPCK) blocked MT-III-induced COX-2 expression and PGE_2_ release. The involvement of NF-*κ*B as the mechanism underlying MT-III-induced upregulation of COX-2 expression was further confirmed by results with inhibition of NF-*κ*B nuclear translocation site by the compound SN50, which markedly reduced MT-III-induced COX-2 expression and PGE_2_ synthesis. Thus, MT-III activates downstream pathways required for upregulation of COX-2 expression through activation of NF-*κ*B. Our data are in agreement with findings that a recombinant group IIA sPLA_2_ induced the activation of NF-*κ*B in the macrophage cell line Raw 264.7 [31]. To our knowledge, this is the first demonstration of the existence of a link between NF-*κ*B and a group IIA sPLA_2_ leading to expression of COX-2 and production of PGE_2_.

Despite various efforts to study in detail the inflammatory mechanisms triggered by group IIA Asp49 sPLA_2_, the signal transduction mechanism is still unclear. In particular, it is not well understood how the signal transduction pathways are started by extracellular MT-III stimuli in peritoneal macrophages, since no receptors or acceptors of group IIA snake venom sPLA_2_ have been described. Since protein kinases are part of the signal transduction pathways which connect inflammatory and other extracellular signals with intracellular responses, such as protein synthesis, we investigated the role of some protein kinases which have been shown to participate in COX-2 upregulation induced by inflammatory and infectious stimuli, such as PKC [21, 23], p38MAPKs [19], and PI3K [21, 22]. Our data demonstrate, for the first time, that a type IIA Asp49 sPLA_2_ from snake venom is able to activate phosphorylation of these kinase proteins in isolated macrophages. Next, by using pharmacological approaches, we investigated the role of these kinases in PGE_2_ release and COX-2 expression. It was found that MT-III effects in macrophages are regulated by specific signaling pathways and that the signaling proteins p38MAPK and PKC are distinctly involved in COX-2 expression, PGE_2_ release, and activation of NF-*κ*B. Our data are consistent with other studies in which activation of p38MAPK is a critical link in inflammation, cytotoxicity, and lipid body formation induced by type IIA sPLA_2_ from both human [32] and snake venoms [26, 35]. In this context, some works confirmed that p38MAPK-NF-*κ*B pathway is an important component of cellular signal transduction, especially in regulating inflammatory genes [36, 37] and that p38MAPK specific inhibitors greatly attenuate NF-*κ*B nuclear translocation [38, 39], COX-2 expression, and PGE_2_ release [40].

Similarly, the observation that production of PGE_2_ and expression of COX-2 via NF-*κ*B in murine macrophages activated by MT-III is dependent on the PKC pathway agrees with other studies in that the groups IIA and VsPLA_2_ activate PKC signaling protein in some cell types [41, 42]. It also agrees with the observation that this protein kinase is required for PGE_2_ biosynthesis, COX-2 expression, and NF-*κ*B activation in both RAW 264.7 cells and mouse peritoneal macrophages upon inflammatory stimuli [43, 44]. In contrast, we showed that blockade of MT-III function with PI3K inhibitor is sufficient to suppress both PGE_2_ production and COX-2 expression but is unable to suppress NF-*κ*B activation. The observation that PI3K is critically involved in MT-III-induced COX-2 and PGE_2_ production is consistent with previous reports that PI3K pathway is recruited for COX-2 expression under different inflammatory conditions [45, 46]. Since the effect of MT-III on COX-2 expression and PGE_2_ release could be explained by an upregulation of *κ*B-dependent transcription in murine macrophages, we hypothesize that some of the signaling pathways activated by MT-III are also exerted through another regulatory element(s), because this sPLA_2_ still induces the activation of NF-*κ*B in the presence of PI3K inhibitor. It is suggested that, besides NF-*κ*B, MT-III leads to the activation of other types of transcription factors. In agreement with this hypothesis, there are reports that PI3K is required for cAMP response element-binding (CREB) or activator protein-1 (AP-1) activation by different stimuli for downstream COX-2 protein synthesis [47, 48]. Although our results have identified selected downstream pathways regulating key steps involved in the biosynthesis of COX-2 expression and PGE_2_ synthesis induced by MT-III, the mechanism of sPLA_2_-IIA-mediated PI3K and other protein kinases activation involved in COX-2 upregulation, remains to be determined.

## 5. Conclusions 

The involvement of distinct pathways mediated by p38MAPK/NF-*κ*B and PKC/NF-*κ*B is essential for MT-III-induced PGE_2_ release via COX-2 protein. Moreover, our results indicate that there is no crosstalk between PI3K phosphorylation and NF-*κ*B activation implicated in MT-III COX-2 expression and PGE_2_ production in our experimental conditions. Taken together, the results presented provide new insights into the mechanisms involved in the production of PGE_2_ through the COX-2 pathway by further defining distinct signaling pathways induced by an Asp49 IIA sPLA_2_ from snake venom.
